# Trends in life expectancy among medical aid beneficiaries and National Health Insurance beneficiaries in Korea between 2004 and 2017

**DOI:** 10.1186/s12889-019-7498-2

**Published:** 2019-08-19

**Authors:** Jinwook Bahk, Hee-Yeon Kang, Young-Ho Khang

**Affiliations:** 1 Department of Public Health, Keimyung University, 1095, Dalgubeol-daero, Dalseo-gu, Daegu, 42601 South Korea; 2 Department of Health Policy and Management, Seoul National University College of Medicine, 103 Daehak-ro, Jongno-gu, Seoul, 03080 South Korea; 3 Institute of Health Policy and Management, Seoul National University Medical Research Center, 103 Daehak-ro, Jongno-gu, Seoul, 03080 South Korea

**Keywords:** Life expectancy, Medical aid, National Health Insurance, Republic of Korea

## Abstract

**Background:**

Medical Aid beneficiaries in Korea are more likely to have poor health status and to receive insufficient healthcare services, but their life expectancy has not been compared with that of National Health Insurance beneficiaries.

**Methods:**

We used the National Health Information Database in Korea to obtain aggregate data on the numbers of population and deaths according to calendar year (2004 to 2017), sex, age group, and insurance eligibility (Medical Aid or National Health Insurance). Between 2004 and 2017, a summed total of 697,503,634 subjects (combining numbers of subjects for 14 years) and 3,536,778 deaths, including 22,417,216 Medical Aid beneficiaries and 499,604 associated deaths, were used to construct annual abridged life tables.

**Results:**

In 2017, the life expectancy of Medical Aid beneficiaries was 70.9 years, while that of National Health Insurance beneficiaries was 83.7 years. Between 2004 and 2017, life expectancy for Medical Aid beneficiaries increased by 8.7 years in men and 6.1 years in women, while life expectancy for National Health Insurance beneficiaries increased by 5.2 years in men and 4.5 years in women. The life expectancy difference between National Health Insurance beneficiaries and Medical Aid beneficiaries was especially great among men across all study periods. The life expectancy difference was 15.8 years for men and 8.9 years for women in 2017.

**Conclusions:**

The life expectancy of Medical Aid beneficiaries was shorter than that of National Health Insurance beneficiaries. The government should implement policies to deliver more adequate health care to Medical Aid beneficiaries.

**Electronic supplementary material:**

The online version of this article (10.1186/s12889-019-7498-2) contains supplementary material, which is available to authorized users.

## Background

In Republic of Korea (hereafter ‘Korea’), the Medical Aid system is a public assistance program that provides healthcare benefits to low-income families, such as those who are eligible through the National Basic Living Security Act. Medical Aid beneficiaries accounted for 2.8% of all Koreans in 2017, while all other Koreans (97.2%) were beneficiaries of National Health Insurance [1]. Medical Aid, along with National Health Insurance, is an important social security system that functions to protect people’s health in Korea. However, despite its goal of solving the medical problems of low-income families, it is difficult to say whether the Medical Aid system is working adequately to that end. Compared to National Health Insurance beneficiaries, several studies have reported that Medical Aid beneficiaries had poor health status and received insufficient medical care. Medical Aid beneficiaries showed higher risks of chronic diseases [2, 3] and suicide [4], lower levels of medication adherence and continuity of treatment [5, 6], and lower effectiveness of behavioral intervention programs [7]. They also were more likely to receive lower-quality medical care [8–11], more vulnerable to environmental conditions [12, 13], and less likely to participate in national screening programs [14, 15]. Consequently, Medical Aid beneficiaries have a higher risk of mortality than National Health Insurance beneficiaries [16]. As far as we are aware, however, no studies have compared life expectancy trends between Medical Aid beneficiaries and National Health Insurance beneficiaries in Korea. The purpose of this study was to estimate and compare life expectancy at birth among Medical Aid beneficiaries and National Health Insurance beneficiaries in Korea between 2004 and 2017 based on whole-population data from the National Health Insurance Service (NHIS).

## Methods

Aggregate data on the numbers of population and deaths according to calendar year (2004 to 2017), sex, age groups (0, 1–4, 5–9, 10–11, …, 85+), and eligibility (Medical Aid or National Health Insurance) were obtained from the National Health Information Database (NHID) provided by the NHIS in Korea [17]. The NHID data cover all Medical Aid beneficiaries and National Health Insurance beneficiaries in Korea except for foreigners. In this study, Medical Aid beneficiaries were defined as recipients of medical benefits as of the first day of the year based on the Medical Care Assistance Act. Medical Aid beneficiaries and National Health Insurance beneficiaries are mutually exclusive groups. National Health Insurance beneficiaries were Korean nationals who reside within the country, excluding those who receive medical benefits, based on National Health Insurance Act. Additional file 1: Table S1 presents the annual numbers of population and deaths according to eligibility during the study period. Between 2004 and 2017, a summed total of 697,503,634 subjects (675,086,418 National Health Insurance beneficiaries and 22,417,216 Medical Aid beneficiaries) and 3,536,778 deaths (3,037,174 National Health Insurance beneficiaries and 499,604 Medical Aid beneficiaries) were analyzed (see Additional file 1: Table S1). The Medical Aid beneficiaries accounted for 2.8–3.7% of all Korean medical security beneficiaries during 2004–2017 (see Additional file 1: Figure S1).

Between 2004 and 2017, annual abridged life tables were constructed using 5-year probabilities of death separately for Medical Aid beneficiaries and National Health Insurance beneficiaries. Standard life table procedures were used to calculate survival rates [18], and the Kannisto-Thatcher method was employed to expand the open-ended age interval of 85+ to estimate the probability of dying for each of the 5-year age groups of 85–89, 90–94, …, 120–124, and 125 + [19]. In addition, an additional analysis was conducted to calculate the life expectancy and life expectancy differences according to the National Health Insurance type and eligibility (see Additional file 1: Table S2).

## Results

**Fig. 1:**
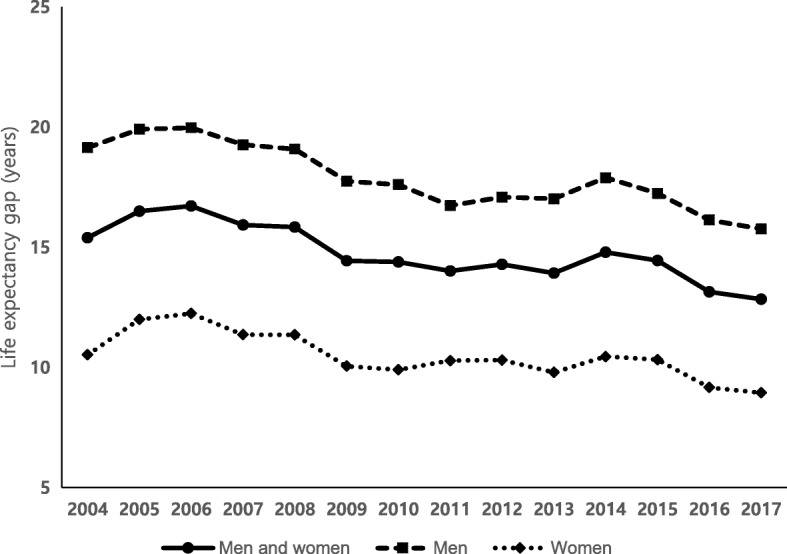
Trends in life expectancy differences between National Health Insurance beneficiaries and Medical Aid beneficiaries by sex from 2004 to 2017

Between 2004 and 2017, life expectancy increased for both National Health Insurance beneficiaries and Medical Aid beneficiaries, but the magnitude of the increase was greater for Medical Aid beneficiaries. During the 13-year study period between 2004 and 2017, life expectancy for Medical Aid beneficiaries increased by 8.7 years in men and 6.1 years in women, respectively, while life expectancy for National Health Insurance beneficiaries increased by 5.2 years in men and 4.5 years in women (Table 1). Therefore, the life expectancy difference decreased from 15.4 years in 2004 to 12.8 years in 2017. The life expectancy difference between National Health Insurance beneficiaries and Medical Aid beneficiaries was larger in men than in women in all calendar years. The life expectancy difference ranged from 20.0 years to 15.8 years (average of 17.9 years during the study period) among men, while the gap ranged from 12.2 years to 8.9 years (average of 10.5 years during the study period) among women (Fig. 1, see Additional file 1: Table S3 for more detailed values). Women showed much higher life expectancies than men among both National Health Insurance beneficiaries and Medical Aid beneficiaries (Table 1). However, the sex difference in life expectancy was much greater among Medical Aid beneficiaries than among National Health Insurance beneficiaries. In 2017, the life expectancy difference between men and women was 12.6 years for Medical Aid beneficiaries, while the difference was 5.8 years for National Health Insurance beneficiaries (Table 1).

**Table 1:** Annual life expectancy among National Health Insurance (NHI) beneficiaries and Medical Aid beneficiaries between 2004 and 2017 by sex

Year	Men and Women		Men		Women	
	NHI	Medical Aid	NHI	Medical Aid	NHI	Medical Aid
2004	78.8	63.4	75.4	56.2	81.9	71.4
2005	79.3	62.8	76.0	56.1	82.2	70.2
2006	79.9	63.2	76.6	56.6	82.8	70.5
2007	80.2	64.3	76.9	57.7	83.0	71.7
2008	80.8	65.0	77.4	58.4	83.7	72.3
2009	81.2	66.7	77.8	60.0	84.1	74.0
2010	81.3	67.0	77.9	60.3	84.2	74.3
2011	81.7	67.7	78.3	61.6	84.6	74.3
2012	81.9	67.6	78.5	61.4	84.8	74.5
2013	82.4	68.5	79.1	62.1	85.3	75.5
2014	82.8	68.0	79.5	61.6	85.5	75.1
2015	83.1	68.7	79.9	62.7	85.8	75.5
2016	83.4	70.3	80.3	64.2	86.1	77.0
2017	83.7	70.9	80.6	64.9	86.4	77.5

## Discussion

The results of this study indicate that the life expectancy of Medical Aid beneficiaries was much shorter than that of National Health Insurance beneficiaries in Korea. The life expectancy gap was 15.8 years among men and 8.9 years among women in 2017. It is possible that poor health determines household poverty, especially among Medical Aid beneficiaries in Korea [20]. People who are not in good health may lose their job and then become poor, making them eligible to receive Medical Aid program benefits. However, it is also possible that other factors, such as socioeconomic disadvantages, discrimination, limited access to health care services, and lower quality of medical services might contribute to the lower life expectancy among Medical Aid beneficiaries than among National Health Insurance beneficiaries. These factors are suspected to create gaps between Medical Aid beneficiaries and National Health Insurance beneficiaries in all areas from prevention to treatment. For example, a smoking cessation program was less effective for Medical Aid beneficiaries and cessation rates were lower for Medical Aid beneficiaries than for National Health Insurance beneficiaries [7]. The participation rates in national screening programs, such as the National Screening Program for Transitional Ages or the National Cancer Screening Program, were lower among Medical Aid beneficiaries than among National Health Insurance beneficiaries [14, 15]. Compared to National Health Insurance beneficiaries, Medical Aid beneficiaries showed lower antihypertensive medication adherence and lower continuity of diabetes care [5, 6]. Medical Aid beneficiaries showed a higher acute appendicitis rupture rate than did National Health Insurance beneficiaries [8]. Among patients with schizophrenia, Medical Aid beneficiaries were less likely to use atypical drugs, which were recommended as the first-line treatment for schizophrenia, than National Health Insurance beneficiaries [9]. Medical Aid patients were more likely to discontinue lung cancer treatment than were high-income patients [10]. Medical Aid beneficiaries are economically and socially vulnerable, and therefore more likely to develop more serious conditions. The liver cancer risk in patients with liver disease was higher among Medical Aid beneficiaries than among National Health Insurance beneficiaries [2]. Medical Aid beneficiaries had a higher suicide rate than National Health Insurance beneficiaries [4]. In addition, the Medical Aid beneficiaries were more vulnerable to environmental conditions. The effect of heat and cold on hospital admissions for acute myocardial infarction and subarachnoid hemorrhage was greater in Medical Aid beneficiaries than in National Health Insurance beneficiaries [12, 13].

The results of this study showed that the gender gap of life expectancy was much greater in Medical Aid beneficiaries than in National Health Insurance beneficiaries, and this pattern was maintained between 2004 and 2017. The result that the sex difference in life expectancy was more prominent among Medical Aid beneficiaries could be partially explained by the alcohol consumption. Prior Korean study showed that alcohol-related conditions such as alcoholic liver disease, suicide, transport accidents, and liver cancer were major contributors to gender differences of life expectancy inequalities by income [21].

In this study, the life expectancy difference between National Health Insurance beneficiaries and Medical Aid beneficiaries decreased over the study period. One possible explanation could be positive effects of the continuing revision of the Medical Care Assistance Act [22]. For example, in 2007, revisions of the Medical Care Assistance Act included a reduction of the support obligors who are responsible for supporting eligible recipients, a ban on deposits when a Medical Aid beneficiary is admitted, and providing priority when a Medical Aid beneficiary is injured. These reforms might have improved the health status of Medical Aid beneficiaries.

It should be noted that we found a similar pattern between the annual percentage of Medical Aid beneficiaries and trends in the life expectancy differences between National Health Insurance beneficiaries and Medical Aid beneficiaries (see Fig. 1 and Additional file 1: Figure S1). The size of recipients of Medical Aid beneficiaries varies from year to year (Additional file 1: Figure S1). The life expectancy differences between National Health Insurance beneficiaries and Medical Aid beneficiaries varies from year to year also (Fig. 1). The various patterns of the two figures are somewhat similar, although they were not perfectly matched. If this was not a coincidence, a potential explanation may relate to the health status of low-income groups not included as Medical Aid beneficiaries. The life expectancy difference was high when the percentage of Medical Aid beneficiaries was high. This might have been because people who were more vulnerable in terms of health risks than National Health Insurance beneficiaries (for example, the second-lowest income bracket) were not fully eligible to receive Medical Aid program benefits, even though their health needs were urgent. This possibility should be examined in a future study exploring the health status of low-income groups who are not Medical Aid beneficiaries. Several US studies have shown positive effects of state Medicaid expansions on mortality reduction, including infant mortality, especially among disadvantaged populations [23–25].

A limitation of this study is that we were not able to distinguish the type of medical benefits, despite the possibility that there might have been differences in mortality patterns between Medical Aid type 1 (for those being incapable of working) and Medical Aid type 2 (for those being capable of working). However, so far as we are aware, this is the first report of life expectancy at birth and its time trends in Medical Aid beneficiaries compared to National Health Insurance beneficiaries using whole-population data in Korea.

## Conclusions

In conclusion, this study showed that Medical Aid beneficiaries had a much lower life expectancy than National Health Insurance beneficiaries between 2004 and 2017. The government should implement more effective policies to reduce these life expectancy differences and to protect the health of Medical Aid beneficiaries.

## Additional file

Additional file 1: **Figure S1.** Annual numbers and percentage of Medical Aid beneficiaries, as of the end of the year, in Korea from 2004 to 2017 (data from Medical Aid statistics by National Health Insurance Service). **Table S1.** Number of population (as of the first day of the year) and deaths according to eligibility. **Table S2.** Life expectancy and life expectancy differences according to type of insurance and eligibility by sex. **Table S3.** Life expectancy differences between National Health Insurance beneficiaries and Medical Aid beneficiaries by sex. (DOCX 97 kb)

## Data Availability

The data that support the findings of this study are available from the National Health Insurance Service in Korea but restrictions apply to the availability of these data, which were used under license for the current study, and so are not publicly available. Data are however available from the authors upon reasonable request and with permission of the National Health Insurance Service in Korea.

## Abbreviations

NHID: National Health Information Database
NHIS: National Health Insurance Service
