# Supplementary material for: Trends in life expectancy among medical aid beneficiaries and National Health Insurance beneficiaries in Korea between 2004 and 2017
Source: BMC Public Health. 2019 Aug 19;19:1137. doi: 10.1186/s12889-019-7498-2 (PMC6701124; doi:10.1186/s12889-019-7498-2)
Supplement: Supplementary file 1 — Figure S1. Annual numbers and percentage of Medical Aid beneficiaries, as of the end of the year, in Korea from 2004 to 2017 (data from Medical Aid statistics by National Health Insurance Service). Table S1. Number of population (as of the first day of the year) and deaths according to eligibility. Table S2. Life expectancy and life expectancy differences according to type of insurance and eligibility by sex. Table S3. Life expectancy differences between National Health Insurance beneficiaries and Medical Aid beneficiaries by sex. (DOCX 97 kb) [file 12889_2019_7498_MOESM1_ESM.docx]

Figure S1. Annual numbers and percentage of Medical Aid beneficiaries, as of the end of the year, in Korea from 2004 to 2017 (data from Medical Aid statistics by National Health Insurance Service)

Table S1. Number of population (as of the first day of the year) and deaths according to eligibility

| Year | Overall | | National Health Insurance | | Medical Aid | |
| --- | --- | --- | --- | --- | --- | --- |
|  | No. of population | No. of deaths | No. of population | No. of deaths | No. of population | No. of deaths |
| Men and Women | | | | | | |
| 2004 | 48,329,061 | 237,419 | 46,905,259 | 206,047 | 1,423,802 | 31,372 |
| 2005 | 48,530,981 | 237,912 | 47,029,371 | 204,060 | 1,501,610 | 33,852 |
| 2006 | 48,737,480 | 236,995 | 47,006,778 | 198,994 | 1,730,702 | 38,001 |
| 2007 | 48,967,709 | 240,047 | 47,159,661 | 201,557 | 1,808,048 | 38,490 |
| 2008 | 49,245,374 | 239,798 | 47,403,267 | 200,905 | 1,842,107 | 38,893 |
| 2009 | 49,519,213 | 240,935 | 47,689,560 | 204,211 | 1,829,653 | 36,724 |
| 2010 | 49,756,545 | 249,930 | 48,093,925 | 214,021 | 1,662,620 | 35,909 |
| 2011 | 49,980,840 | 252,191 | 48,324,794 | 215,822 | 1,656,046 | 36,369 |
| 2012 | 50,228,255 | 262,277 | 48,634,986 | 225,154 | 1,593,269 | 37,123 |
| 2013 | 50,455,808 | 259,431 | 48,961,585 | 225,323 | 1,494,223 | 34,108 |
| 2014 | 50,658,369 | 262,816 | 49,212,587 | 227,845 | 1,445,782 | 34,971 |
| 2015 | 50,855,927 | 269,976 | 49,437,004 | 235,119 | 1,418,923 | 34,857 |
| 2016 | 51,042,438 | 271,023 | 49,524,158 | 236,893 | 1,518,280 | 34,130 |
| 2017 | 51,195,634 | 276,028 | 49,703,483 | 241,223 | 1,492,151 | 34,805 |
| Sum | 697,503,634 | 3,536,778 | 675,086,418 | 3,037,174 | 22,417,216 | 499,604 |
| Men | | | | | | |
| 2004 | 24,243,142 | 130,834 | 23,637,963 | 115,706 | 605,179 | 15,128 |
| 2005 | 24,338,398 | 130,372 | 23,695,956 | 114,007 | 642,442 | 16,365 |
| 2006 | 24,438,669 | 130,038 | 23,686,040 | 111,326 | 752,629 | 18,712 |
| 2007 | 24,549,015 | 131,617 | 23,758,727 | 112,491 | 790,288 | 19,126 |
| 2008 | 24,681,837 | 132,347 | 23,876,217 | 113,179 | 805,620 | 19,168 |
| 2009 | 24,814,033 | 133,287 | 24,011,827 | 115,339 | 802,206 | 17,948 |
| 2010 | 24,922,943 | 138,129 | 24,195,480 | 120,658 | 727,463 | 17,471 |
| 2011 | 25,023,245 | 139,216 | 24,299,816 | 121,852 | 723,429 | 17,364 |
| 2012 | 25,136,066 | 143,509 | 24,436,925 | 125,925 | 699,141 | 17,584 |
| 2013 | 25,237,439 | 141,885 | 24,582,055 | 125,684 | 655,384 | 16,201 |
| 2014 | 25,327,681 | 143,549 | 24,692,284 | 126,767 | 635,397 | 16,782 |
| 2015 | 25,415,837 | 146,065 | 24,786,628 | 129,277 | 629,209 | 16,788 |
| 2016 | 25,498,033 | 146,293 | 24,820,487 | 129,393 | 677,546 | 16,900 |
| 2017 | 25,563,384 | 148,139 | 24,893,770 | 131,104 | 669,614 | 17,035 |
| Sum | 349,189,722 | 1,935,280 | 339,374,175 | 1,692,708 | 9,815,547 | 242,572 |
| Women | | | | | | |
| 2004 | 24,085,919 | 106,585 | 23,267,296 | 90,341 | 818,623 | 16,244 |
| 2005 | 24,192,583 | 107,540 | 23,333,415 | 90,053 | 859,168 | 17,487 |
| 2006 | 24,298,811 | 106,957 | 23,320,738 | 87,668 | 978,073 | 19,289 |
| 2007 | 24,418,694 | 108,430 | 23,400,934 | 89,066 | 1,017,760 | 19,364 |
| 2008 | 24,563,537 | 107,451 | 23,527,050 | 87,726 | 1,036,487 | 19,725 |
| 2009 | 24,705,180 | 107,648 | 23,677,733 | 88,872 | 1,027,447 | 18,776 |
| 2010 | 24,833,602 | 111,801 | 23,898,445 | 93,363 | 935,157 | 18,438 |
| 2011 | 24,957,595 | 112,975 | 24,024,978 | 93,970 | 932,617 | 19,005 |
| 2012 | 25,092,189 | 118,768 | 24,198,061 | 99,229 | 894,128 | 19,539 |
| 2013 | 25,218,369 | 117,546 | 24,379,530 | 99,639 | 838,839 | 17,907 |
| 2014 | 25,330,688 | 119,267 | 24,520,303 | 101,078 | 810,385 | 18,189 |
| 2015 | 25,440,090 | 123,911 | 24,650,376 | 105,842 | 789,714 | 18,069 |
| 2016 | 25,544,405 | 124,730 | 24,703,671 | 107,500 | 840,734 | 17,230 |
| 2017 | 25,632,250 | 127,889 | 24,809,713 | 110,119 | 822,537 | 17,770 |
| Sum | 348,313,912 | 1,601,498 | 335,712,243 | 1,344,466 | 12,601,669 | 257,032 |

Table S2. Life expectancy and life expectancy differences according to type of insurance and eligibility by sex

| Year | Life expectancy | | | | Life expectancy differences | | | | |
| --- | --- | --- | --- | --- | --- | --- | --- | --- | --- |
|  | Overall [A] | Employed [B] | Self-employed [C] | Medical Aid [D] | [A-B] | [A-C] | [A-D] | [B-D] | [C-D] |
| Men and Women | | | | | | | | | |
| 2004 | 78.3 | 79.6 | 78.0 | 63.4 | -1.3 | 0.3 | 14.8 | 16.2 | 14.5 |
| 2005 | 78.7 | 80.0 | 78.5 | 62.8 | -1.3 | 0.2 | 15.9 | 17.2 | 15.7 |
| 2006 | 79.1 | 80.4 | 79.2 | 63.2 | -1.3 | -0.1 | 15.9 | 17.2 | 16.0 |
| 2007 | 79.4 | 80.7 | 79.4 | 64.3 | -1.3 | 0.0 | 15.2 | 16.5 | 15.2 |
| 2008 | 80.0 | 81.4 | 79.9 | 65.0 | -1.3 | 0.1 | 15.1 | 16.4 | 15.0 |
| 2009 | 80.5 | 81.8 | 80.1 | 66.7 | -1.3 | 0.3 | 13.8 | 15.1 | 13.4 |
| 2010 | 80.7 | 82.0 | 80.3 | 67.0 | -1.3 | 0.4 | 13.7 | 15.0 | 13.3 |
| 2011 | 81.1 | 82.3 | 80.6 | 67.7 | -1.2 | 0.5 | 13.4 | 14.6 | 12.9 |
| 2012 | 81.3 | 82.5 | 80.8 | 67.6 | -1.2 | 0.5 | 13.6 | 14.9 | 13.2 |
| 2013 | 81.9 | 83.1 | 81.1 | 68.5 | -1.2 | 0.7 | 13.4 | 14.6 | 12.6 |
| 2014 | 82.2 | 83.4 | 81.4 | 68.0 | -1.2 | 0.8 | 14.2 | 15.4 | 13.4 |
| 2015 | 82.5 | 83.7 | 81.8 | 68.7 | -1.2 | 0.8 | 13.9 | 15.1 | 13.1 |
| 2016 | 83.0 | 84.1 | 82.0 | 70.3 | -1.1 | 0.9 | 12.7 | 13.8 | 11.7 |
| 2017 | 83.2 | 84.4 | 82.2 | 70.9 | -1.2 | 1.1 | 12.4 | 13.5 | 11.3 |
| Men | | | | | | | | | |
| 2004 | 74.7 | 76.5 | 74.2 | 56.2 | -1.9 | 0.5 | 18.4 | 20.3 | 17.9 |
| 2005 | 75.2 | 77.0 | 74.9 | 56.1 | -1.8 | 0.3 | 19.1 | 20.9 | 18.8 |
| 2006 | 75.7 | 77.4 | 75.6 | 56.6 | -1.7 | 0.0 | 19.0 | 20.7 | 19.0 |
| 2007 | 76.0 | 77.6 | 75.9 | 57.7 | -1.6 | 0.1 | 18.3 | 20.0 | 18.3 |
| 2008 | 76.5 | 78.2 | 76.2 | 58.4 | -1.7 | 0.3 | 18.2 | 19.9 | 17.9 |
| 2009 | 76.9 | 78.6 | 76.5 | 60.0 | -1.7 | 0.5 | 16.9 | 18.6 | 16.5 |
| 2010 | 77.1 | 78.8 | 76.5 | 60.3 | -1.6 | 0.6 | 16.8 | 18.5 | 16.2 |
| 2011 | 77.5 | 79.1 | 76.9 | 61.6 | -1.5 | 0.7 | 16.0 | 17.5 | 15.3 |
| 2012 | 77.8 | 79.2 | 77.2 | 61.4 | -1.5 | 0.6 | 16.3 | 17.8 | 15.8 |
| 2013 | 78.4 | 79.9 | 77.5 | 62.1 | -1.5 | 0.9 | 16.3 | 17.8 | 15.4 |
| 2014 | 78.8 | 80.3 | 77.9 | 61.6 | -1.5 | 0.9 | 17.2 | 18.7 | 16.3 |
| 2015 | 79.2 | 80.6 | 78.4 | 62.7 | -1.4 | 0.9 | 16.6 | 18.0 | 15.7 |
| 2016 | 79.7 | 81.0 | 78.8 | 64.2 | -1.3 | 0.8 | 15.5 | 16.8 | 14.7 |
| 2017 | 80.0 | 81.4 | 79.0 | 64.9 | -1.4 | 1.1 | 15.1 | 16.5 | 14.1 |
| Women | | | | | | | | | |
| 2004 | 81.5 | 82.4 | 81.4 | 71.4 | -0.9 | 0.1 | 10.1 | 11.0 | 10.0 |
| 2005 | 81.7 | 82.6 | 81.7 | 70.2 | -0.9 | 0.0 | 11.5 | 12.4 | 11.5 |
| 2006 | 82.1 | 83.0 | 82.4 | 70.5 | -0.9 | -0.2 | 11.6 | 12.5 | 11.8 |
| 2007 | 82.5 | 83.4 | 82.5 | 71.7 | -0.9 | -0.1 | 10.8 | 11.7 | 10.8 |
| 2008 | 83.1 | 84.0 | 83.2 | 72.3 | -0.9 | -0.1 | 10.7 | 11.7 | 10.8 |
| 2009 | 83.5 | 84.5 | 83.3 | 74.0 | -1.0 | 0.2 | 9.5 | 10.5 | 9.3 |
| 2010 | 83.7 | 84.6 | 83.4 | 74.3 | -0.9 | 0.3 | 9.4 | 10.3 | 9.1 |
| 2011 | 84.1 | 85.0 | 83.8 | 74.3 | -0.9 | 0.3 | 9.8 | 10.7 | 9.5 |
| 2012 | 84.2 | 85.2 | 83.8 | 74.5 | -1.0 | 0.4 | 9.8 | 10.8 | 9.4 |
| 2013 | 84.8 | 85.7 | 84.3 | 75.5 | -0.9 | 0.5 | 9.3 | 10.2 | 8.9 |
| 2014 | 85.0 | 86.0 | 84.5 | 75.1 | -0.9 | 0.5 | 10.0 | 10.9 | 9.5 |
| 2015 | 85.3 | 86.3 | 84.8 | 75.5 | -0.9 | 0.6 | 9.9 | 10.8 | 9.3 |
| 2016 | 85.8 | 86.7 | 84.8 | 77.0 | -0.9 | 0.9 | 8.8 | 9.7 | 7.9 |
| 2017 | 86.0 | 87.0 | 85.1 | 77.5 | -0.9 | 1.0 | 8.6 | 9.5 | 7.6 |

Table S3. Life expectancy differences between National Health Insurance beneficiaries and Medical Aid beneficiaries by sex

| Year | Men and women | Men | Women |
| --- | --- | --- | --- |
| 2004 | 15.4 | 19.1 | 10.5 |
| 2005 | 16.5 | 19.9 | 12.0 |
| 2006 | 16.7 | 20.0 | 12.2 |
| 2007 | 15.9 | 19.3 | 11.4 |
| 2008 | 15.8 | 19.1 | 11.4 |
| 2009 | 14.4 | 17.7 | 10.1 |
| 2010 | 14.4 | 17.6 | 9.9 |
| 2011 | 14.0 | 16.7 | 10.3 |
| 2012 | 14.3 | 17.1 | 10.3 |
| 2013 | 13.9 | 17.0 | 9.8 |
| 2014 | 14.8 | 17.9 | 10.5 |
| 2015 | 14.4 | 17.2 | 10.3 |
| 2016 | 13.1 | 16.1 | 9.2 |
| 2017 | 12.8 | 15.8 | 8.9 |
